# AMH as a Predictor of Follicle Turnover, Embryo Quality, and Pregnancy Outcomes: A Retrospective Analysis

**DOI:** 10.3390/biomedicines13122875

**Published:** 2025-11-25

**Authors:** Fatma Kılıç Hamzaoğlu, Serdar Dilbaz, Runa Özelçi, Onur Kaya, Emine Utlu Özen

**Affiliations:** 1 Department of Gynecology and Obstetrics, Faculty of Medicine, Necmettin Erbakan University, Konya 42090, Turkey; 2 Department of Gynecology and Obstetrics, University of Health Sciences, Etlik Zübeyde Hanım Women’s Health Training and Research Hospital, Ankara 06620, Turkey

**Keywords:** anti-Müllerian hormone, intracytoplasmic sperm injection, ovarian reserve, embryo quality

## Abstract

**Background/Objectives**: Infertility affects ~10–15% of couples of reproductive age, and assisted reproductive technologies (ARTs) increasingly rely on biomarkers to individualize care. This study aimed to investigate the relationship between serum anti-Müllerian hormone (AMH) levels and embryo quality and pregnancy outcomes in patients undergoing intracytoplasmic sperm injection (ICSI). We specifically evaluated whether AMH predicts embryo competence and clinical pregnancy beyond its established role in ovarian reserve assessment. **Methods**: This retrospective study included 1990 women undergoing ICSI between 2010 and 2023, categorized into three groups (G1–G3) based on antral follicle count (AFC). Embryo morphology was graded using ASEBIR criteria with prospectively maintained lab SOPs. Clinical, embryological, and pregnancy parameters were compared using non-parametric tests, ROC analysis, and logistic regression. The primary outcome was clinical pregnancy; biochemical pregnancy was recorded; live birth was not available and is acknowledged as a limitation. **Results**: Higher AMH levels correlated with increased AFC and oocyte yield (all *p* < 0.001) but showed no clinically meaningful association with high-grade embryos (Grade 1–2) or pregnancy. ROC analyses demonstrated limited discrimination for AMH (AUC ≈ 0.49); by contrast, age and FSH showed modest discrimination (AUC 0.56 and 0.55, respectively), and embryo-level features (pronuclear count, Grade 1 and Grade 3 counts) were statistically significant yet of limited clinical utility (AUCs near 0.5) **Conclusions**: AMH is robust for ovarian reserve and response prediction but is a weak predictor of embryo morphology and clinical pregnancy. Outcome prediction in ART should integrate age, FSH, and embryo morphology (and, where available, sperm quality) rather than AMH alone. Prospective, multicenter studies with live birth as the primary endpoint are warranted.

## 1. Introduction

Infertility remains a prevalent health problem, affecting approximately 10–15% of couples of reproductive age [1]. With the introduction of assisted reproductive techniques (ARTs) such as in vitro fertilization (IVF) and intracytoplasmic sperm injection (ICSI), new avenues have opened for couples with infertility issues. Clinical decision-making increasingly depends on biomarkers that forecast ovarian response and treatment prognosis. An essential factor in predicting success lies within the markers of ovarian reserves. Of these, anti-Müllerian hormone (AMH) has emerged as one of the key indicators for evaluating the ovarian reserve and for predicting the response to controlled ovarian stimulation [2]. Remotely secreted by the theca and granulosa cells of preantral and small antral follicles, AMH levels decrease in a linear fashion with advancing age and become undetectable during menopause [3]. Due to its constancy through the menstrual cycle, AMH is well known to clinically be an indicator of ovarian activity in ART cycles [4]. Most importantly, the clinically observable anti-Müllerian hormone does indicate the female component of the ovarian reserve. However, in the case of ICSI fertilization and embryonic development, underutilized male determinants such as sperm shape, sperm DNA quality, and even oocyte competence take center stage and may mask the true potential of AMH [5,6].

Serum levels of AMH are often used to assess the number of oocytes that can be retrieved during ART cycles, although the association with oocyte and embryo quality is still contested. Some studies put forward that AMH levels are variables that have a positive association with the quality of the developed embryos. In comparison, other studies suggest that AMH levels have no markedly predictive value [7,8]. Embryo processing in ART requires substantial attention and various scoring systems, including ASEBIR criteria, aim to quantify the embryos for assessing their implantation possibilities [9]. Regardless of its established capability of predicting the ovarian reserve using AMH levels, its influence on embryos and pregnancy outcomes remains uncertain. The disparity in results indicates that more research is needed to understand if AMH levels can be applied for more than evaluating ovarian reserve in relation to predicting embryo viability and chances for clinical pregnancy. The current literature does not reach a conclusion about the accuracy of the assessment of the value of AMH on the quality of the embryo and the clinical outcomes of the pregnancy. Several studies have indicated an association between higher levels of AMH and increased success rates in pregnancy [10,11], while other studies found no such connection [7,12].

This inconsistency may come from differences in study population characteristics, protocols for ovarian stimulation, systems of grading embryos, or even uncontrolled biases like male infertility issues and lab environment factors [13,14].

With the development of ICSI, some recent studies have attempted to examine the correlation of AMH concentration and ICSI cycle outcomes, but the results have not been consistent. For some, there seems to be a link between higher AMH levels and an increase in clinical pregnancy rates [10,11] while for others, no relationship was found [12]. Moreover, the quality of sperm, especially its morphology, has been recognized as having an important role in embryo development and pregnancy, which amplifies the complexity of understanding AMH’s contribution to the results of ART procedures [5]. Such complexity emphasizes the importance of having an integrated investigation tackling the various elements that might influence the quality of embryos and pregnancy results in ART cycles. The premise for this research is that AMH, despite being a recognized indicator of ovarian reserve and number of oocytes retrieved, fails to provide reliable estimates for the quality of embryo and occurrence of pregnancy in ICSI cycles.

This study aims to determine how serum AMH concentrations impact embryo quality and pregnancy results in patients undergoing ICSI. The objective is to assess if AMH levels can indicate not only an ovarian reserve but also the embryo’s potential to develop, thus broadening the role of AMH in clinical practice. Optimizing selection criteria and protocols to improve reproductive outcomes requires a thorough understanding of the dynamics between these factors. These results could facilitate more customized treatment plans in ART by enabling clinicians to adjust treatment approaches according to serum AMH levels and improve odds of achieving successful pregnancies.

## 2. Materials and Methods

The present study aimed to be a retrospective clinical study focused on the impact serum anti-Müllerian hormone (AMH) concentration has on embryo quality and pregnancy results in patients who underwent intracytoplasmic sperm injection (ICSI) was conducted. Study design and setting. Single-center retrospective cohort (1 April 2010–1 April 2023) conducted at University of Health Sciences, Etlik Zübeyde Hanım Women’s Health Training and Research Hospital (Approval Number: 04/02, Date: 25 April 2023). The study followed institutional ART SOPs throughout the period.

### 2.1. Study Population

The study included patients who underwent ICSI between 1 April 2010 and 1 April 2023 as part of an assisted reproductive technology (ART) program. Population and grouping. Patients undergoing ICSI were classified by AFC: poor (0–5), normo (5–15), hyper (>15). Inclusion: women < 40 years, ICSI with embryo transfer during the period. Exclusion: isolated severe male factor (e.g., total motile sperm count < 1 million) and age ≥ 40. Standard semen parameters were recorded but not modeled; this is acknowledged as a limitation. Women < 40 years undergoing ICSI with embryo transfer between 1 April 2010 and 1 April 2023 were included; isolated severe male factor (e.g., total motile sperm <1.0 × 10^6^) and age ≥ 40 were excluded.

We detail ASEBIR training/SOPs over 2010–2023 to mitigate grading drift. We explicitly interpret ROC metrics, noting that AUCs near/below 0.5 lack clinical value even if *p* < 0.05, addressing the reviewer’s concern on confusing ROC statements.

### 2.2. Inclusion and Exclusion Criteria

Patients included in the study were women under 40 years of age who had undergone ICSI and embryo transfer within the specified study period. Patients with controlled ovarian stimulation performed solely due to male factor infertility and those above 40 years of age were excluded. While the study excluded patients with isolated male factor infertility, controlling for covariates such as sperm morphology, concentration, and motility was not carried out based on the semen parameters analyzed. This was a limitation since the quality of sperm, even in cycles utilizing ICSI, can greatly impact fertilization, embryo development, and the subsequent chances of achieving pregnancy.

### 2.3. Data Collection and Study Parameters

Retrospective data were collected from patient medical records, including demographic, clinical, laboratory, and embryological parameters. Key variables recorded were:

- Demographic and clinical parameters: Age, body mass index (BMI), AMH and follicle-stimulating hormone (FSH) levels, and AFC.
- Ovarian stimulation parameters: Total gonadotropin dose administered during ovarian stimulation.
- Oocyte and embryo parameters: Number of retrieved oocytes, number of mature oocytes, number of ICSI-inseminated oocytes, oocyte quality index, pronuclear count, number of embryos transferred, number of blastocysts, and embryo grading based on ASEBIR criteria. Embryo quality was evaluated with the ASEBIR (Asociación para el Estudio de la Biología de la Reproducción) morphological classification’s Grade 1 criterion, which requires symmetric blastomeres, lack of multinucleation, and less than ten percent fragmentation. Grade 2 is characterized by slight asymmetry in blastomeres and 10- to 25-percent fragmentation. Grade 3 includes embryos with pronounced asymmetry, greater than 25% fragmentation, slight multinucleation, while Grade 4 embryos are classified as low quality because of high (>50%) fragmentation or abnormal morphology.
- Pregnancy outcomes: Biochemical pregnancy and clinical pregnancy rates.

### 2.4. Embryo Transfer and Endometrial Preparation

Embryo grading standardization. Embryo morphology was graded per ASEBIR (3rd ed.) using a lab-wide SOP. All embryologists underwent initial training (2010 roll-out) with annual refreshers; inter-observer alignment sessions were performed quarterly, and any manual/criteria updates were documented in the SOP log. Day-3/Day-5 transfer decisions followed clinical and developmental criteria.

Endometrial preparation. Fresh or HRT-prepared FET cycles were managed per SOP (estradiol oral/transdermal; vaginal progesterone once endometrium ≥ 7 mm).

Outcomes. Primary: clinical pregnancy (ultrasound-confirmed). Secondary: biochemical pregnancy. Live birth and miscarriage were not systematically available and are listed as limitations.

### 2.5. Statistical Analysis

Statistical analyses were conducted using SPSS (Statistical Package for the Social Sciences) version 22. The normality of numerical variables was assessed using the Kolmogorov–Smirnov and Shapiro–Wilk tests.

For comparative analyses, Mann–Whitney U and Kruskal–Wallis tests were used for non-normally distributed variables, while ANOVA was applied to normally distributed variables. Receiver Operating Characteristic (ROC) analysis was performed to evaluate the diagnostic performance of clinical parameters in predicting ART outcomes, with area under the curve (AUC), sensitivity, and specificity values reported. Additionally, univariate logistic regression analysis was conducted using the Enter method to assess the impact of continuous variables on pregnancy outcomes, with odds ratios (OR) and 95% confidence intervals (CI) calculated. A *p*-value < 0.05 was considered statistically significant for all analyses. To minimize confounding factors, patients with severe male factor infertility (e.g., total motile sperm count < 1 million) were excluded from the analysis. However, routine semen quality parameters were not included in the regression models, which constitutes a limitation.

## 3. Results

The study was completed with 1990 patients who underwent IVF treatment. The patients’ age, BMI, AMH, FSH, antral follicle count (AFC), total gonadotropin dose administered for ovulation induction, number of retrieved oocytes, number of mature oocytes, number of oocytes subjected to ICSI, oocyte quality index, pronuclear count, number of embryos transferred, number of Grade 1 embryos, Grade 2 embryos, Grade 3 embryos, Grade 4 embryos, and number of blastocysts are presented in Table 1.

Patients were classified based on their antral follicle count as follows: 0–5 as poor responders, 5–15 as normo responders, and above 15 as hyper responders. In the hyper responder group, age and FSH levels were found to be lower compared to the other groups (*p* < 0.001 and *p* < 0.001), while AMH and AFC values were higher (*p* < 0.001 and *p* < 0.001). Additionally, the BMI of the normo responder group was lower than that of both the poor responder and hyper responder groups (*p* < 0.001) (Table 2).

The number of retrieved oocytes, mature oocytes, ICSI-performed oocytes, pronuclear oocytes, and embryo transfers in the hyper responder group were significantly higher compared to the other groups (*p* < 0.001). However, the total medication dose administered for ovulation induction in the hyper responder group was found to be lower than in the other groups (*p* < 0.001). Additionally, the oocyte quality index in the normo responder group was higher than that in the poor responder group (*p* = 0.012) (Table 3).

Patients who achieved pregnancy after IVF treatment had significantly lower age and FSH levels (*p* < 0.001 and *p* < 0.001). No significant difference was noted in the proportions of blastocyst transfers or the day of embryo transfer (Day 3 versus Day 5) among the AMH groups, which could have been factors to testes embryo competence though this was not a primary focus of the study. However, AMH, AFC, number of retrieved oocytes, number of mature oocytes, number of ICSI oocytes, oocyte quality index, pronuclear count, number of embryos transferred, number of Grade 1 embryos, and number of Grade 3 embryos were significantly higher in those who became pregnant (*p* < 0.001) (Table 4).

As serum AMH levels increased, age, BMI, total gonadotropin dose administered for ovulation induction, and the number of Grade 3 embryos decreased (*p* < 0.001, *p* < 0.001, *p* < 0.001, *p* < 0.001, and *p* = 0.048, respectively). In contrast, FSH levels, AFC, number of retrieved oocytes, number of mature oocytes, number of oocytes subjected to ICSI, pronuclear count, number of embryos transferred, and number of blastocysts increased (*p* < 0.001) (Table 5).

The patients’ BMI, AMH, AFC, number of retrieved oocytes, number of mature oocytes, number of oocytes subjected to ICSI, oocyte quality index, number of embryos transferred, number of Grade 2 embryos, number of Grade 4 embryos, and number of blastocysts did not exhibit statistically significant diagnostic performance in predicting IVF treatment outcomes. Despite this, AMH did positively correlate with the number of blastocysts (r = 0.142, *p* < 0.001) while weakly and not significantly associating with high grade (1 and 2) embryos. Correlationally, it is notable that while the relationship of AMH to Grade 3 embryos (r = −0.056, *p* = 0.048) was weak, it still held significance, and was inversely proportional, suggesting a recruitment of suboptimal follicles in high-AMH responding women with a disproportionate recruitment of suboptimal follicles. This requires exploring further in future studies examining if a high ovarian reserve translates to lower competent embryos warranting better embryo competence.

**Figure 1:**
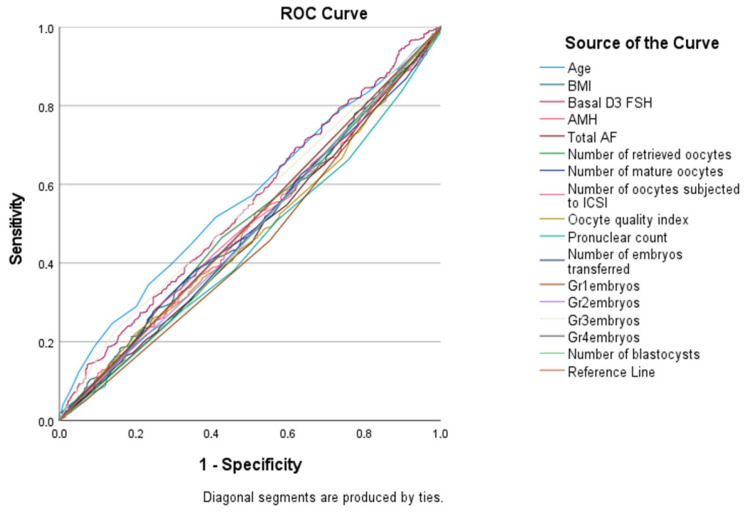
ROC Analysis of Patients’ Clinical, Oocyte, and Embryo Characteristics in Predicting IVF Treatment Outcome.

However, age, FSH levels, pronuclear count, number of Grade 1 embryos, and number of Grade 3 embryos demonstrated statistically significant diagnostic performance (*p* < 0.001, *p* = 0.015, *p* = 0.012, *p* = 0.011, and *p* = 0.036, respectively) (Table 6 and Figure 1). Most AUC values for evaluated parameters were close to 0.5, indicating limited or no predictive utility for pregnancy outcomes. Notably, some parameters with *p* < 0.05 but AUC < 0.5 performed significantly worse than random classification, underscoring their lack of clinical applicability as prognostic tools.

Each unit decrease in age statistically significantly increases the likelihood of a positive IVF outcome by 0.062 times (*p* = 0.001). Similarly, each unit decrease in FSH level statistically significantly increases the likelihood of a positive IVF outcome by 0.072 times (*p* = 0.001). Conversely, each unit increase in the number of Gr1 embryos statistically significantly increases the likelihood of a positive IVF outcome by 0.372 times (*p* = 0.001). Additionally, each unit increase in the number of Gr3 embryos statistically significantly increases the likelihood of a positive IVF outcome by 0.499 times (*p* = 0.002) (Table 7).

The odds ratios (ORs) from the logistic regression analysis were statistically significant, but their impact was rather small. For example, the OR of 0.062 attributed to age means that each year increase in age will increase the odds of getting pregnant by roughly 6.2%, which has some reasonable value in practice but is not strongly suggestive by itself. Also, the impact of every additional Grade 1 embryo (OR = 0.372) indicates that there is 37.2% greater chance of attaining pregnancy, which demonstrates the value of embryo morphology as a more powerful predictor than AMH.

In a group of patients with low AMH levels (<1.0 ng/mL), pregnancy was still possible, though total oocyte yield was lower than average, in a few cycles signifying that with proper stimulation and individualized protocols, success is realistic. These results validate that AMH should not be the only exclusion criteria, and more flexible strategies like modified stimulation of the ovaries, dual stimulations (DuoStim), or pooling embryos may increase possibilities for this population.

## 4. Discussion

The focus of this research was to look into the relationship of serum levels of anti-Müllerian hormones (AMH) and the quality of embryos along with pregnancy results for patients undergoing intracytoplasmic sperm injection (ICSI). The results noted the sophisticated interactions among markers of ovarian reserve, outcomes of oocyte retrievals, and the subsequent embryo development, reinforcing previous positing of conflicting evidence regarding the predictive capabilities of AMH within assisted reproductive technologies (ART).

In the present study we observed that higher levels of AMH increased AFC along with the total number of oocytes, mature oocytes, and oocytes that were inseminated into ICSI, which supports the previous research [2,3]. As noted, AMH levels had an inverse relation with age and FSH levels which is consistent with the accepted notion pertaining to being a marker of reserve ovarian function. Other authors developed similar conclusions which posited that AMH is an efficient indicator of the responsiveness of ovaries to stimulation during a controlled ovarian hyperstimulation [4]. Regardless, it was noted that AMH had a strong impact even though the value was determined to be predictive of the number of oocytes retrieved; its impact on the quality of embryos and possibility of pregnancy remains undetermined. Other recent reviews determined that AMH provided meaningful information pertaining to the reserve of ovaries but is not consistent when estimating the potential a woman possesses regarding fertilization, implantation along with other confounding factors like male infertility, quality of sperms and other variables add on [6].

While AMH was related to the increased number of oocytes retrieved, it did not correlate to the quality of the embryos. Notably, we found a weak yet statistically significant negative association between AMH and Grade 3 embryos, which might indicate increased recruitment of less competent follicles in high-AMH patients, possibly due to polycystic ovary morphology. However, this result requires confirmation. We did not observe any significant relationship between AMH levels and high-quality embryos (Grade 1 and Grade 2 embryos), which was also observed by Smeenk et al. when stating that although AMH levels seem to predict the ovarian response, they do not affect the quality or quantity of embryos produced or the chances of pregnancy [7]. Hafizi et al. also reported no relationship between AMH and embryo morphology in patients undergoing ICSI in Malaysia [12]. In contrast, some studies have noted a positive relationship between AMH and embryos maturation in those found to have adequate ovarian reserve [8,10]. The fact that oocyte quantity and quality appear to be governed by several factors other than AMH, such as the age of the patient, stimulation of the ovaries, and the quality of the sperm, together with the conflicting evidence provided by various studies suggests that there is no consensus in the research.

Aside from the issue of endocrine parameters for specific patients, the type of controlled ovarian stimulation protocol, gonadotropin dosage, and conditions of the embryology lab, such as the type of culture media used and the incubator’s environment, also highly impact an embryo’s morphology and developmental competence [6,15]. Discrepancies in these protocols among institutions may in part explain the conflicting associations between AMH and embryo quality. Importantly, stimulation regimens employing GnRH antagonists, as compared to agonists, will produce different responses in follicle responsive rate and oocyte cyst maturation height, which in turn affects fertilization and embryo grading [13]. As noted by Tian et al., many factors such as AMH levels and structurally objective considerations like embryological and laboratory methods underscore the need to analyze IVF/ICSI cycles from an integrated perspective [13].

Our research noted increased pregnancy rates in patients with elevated AMH levels which is also the case with Park et al. and Umarsingh et al. [10,11]. In these studies, a positive association of AMH levels and clinical pregnancy rates was especially noted in IVF/ICSI cycles in women over 40 years. However, in our study using ROC analysis to evaluate predictive factors for pregnancy success, we found that AMH did not demonstrate adequate diagnostic value regarding the outcomes of IVF. Our ROC analysis showed that AMH, AFC, and most oocyte parameters did not demonstrate meaningful diagnostic performance (AUC ≈ 0.5). Parameters such as pronuclear count and embryo grades that showed *p* < 0.05 but low AUC values should not be interpreted as predictive; rather, they may indicate statistical significance without clinical utility. In contrast, significant determinants of successful pregnancies were older age, higher FSH levels, greater pronuclear count, and an increased number of Grade 1 and Grade 3 embryos. These findings were similar to those of Zhou et al. who stressed the importance of sperm morphology and embryo development in determining the success of ART procedures [5]. These findings stress that while AMH provides an estimation of the ovarian reserve and responsiveness to pharmacologic stimulation, several embryonic and maternal determinants influence pregnancy outcomes. Zhu et al. highlighted that additional treatments, like melatonin, given to patients with repeated IVF/ICSI failures may improve embryo quality and pregnancy outcomes, indicating that success in ART cycles cannot be attributed solely to AMH [16].

The results of this study add to the previously established predictive value of AMH in ovarian reserve, while also noting the risks associated with using this measure as the only determinant for embryo quality or likelihood of successful pregnancy. Integrating age along with FSH, grading of the embryos, and further assessment of sperm may yield better predictions on the success of ART [1]. In practice, AMH remains useful when stratifying and counseling patients regarding their anticipated oocyte yield, but becomes less reliable for determining viability in embryos or predicting pregnancy odds without supplementary markers of relative value. When AMH is low, certain individual protocols may be more effective—DuoStim, minimal stimulation IVF, or pooling of embryos across cycles—especially if associated with diminished ovarian reserve [14]. In addition, some treatments, like melatonin, may help improve the quality of the embryos in patients who have had failed IVF/ICSI cycles [16], and could be especially relevant for those who are low responders. Additionally, the absence of sperm quality data (morphology, motility, DNA fragmentation) and long-term pregnancy outcomes such as live birth or miscarriage limits the comprehensiveness of our findings. These factors are known to influence embryo development and implantation and should be included in future studies to more accurately delineate the predictive role of AMH. This ambiguity concerning AMH and embryo quality highlights the importance of further prospective research focused on the molecular mechanisms associated with oocyte competence.

Furthermore, examining the effects of stimulation protocols, culture conditions and genetics on embryo development will improve understanding of how AMH affects reproductive outcomes. Chen et al. highlight the use of multiple predictive markers for patients with low ovarian reserve and how tailoring these strategies can maximize IVF treatment success [14].

### Limitations

This study’s single-center design limits external validity. Detailed sperm quality parameters (morphology, motility, DNA fragmentation) were unavailable and not modeled, despite their known relevance to embryo development. We did not perform subgroup analyses by transfer day (Day 3 vs. blastocyst). Live birth and miscarriage outcomes were not systematically recorded; conclusions therefore pertain to clinical pregnancy. Additional protocol and laboratory environment variables (e.g., media, incubator systems) and patient-level characteristics were not captured in this retrospective dataset. Collectively, these constraints underscore the need for prospective, multicenter studies incorporating male-factor data and long-term outcomes to delineate the clinical utility of AMH beyond ovarian reserve assessment.

Live birth and miscarriage outcomes were also unavailable; therefore, our conclusions are limited to clinical pregnancy as the primary outcome. This restricts the long-term interpretation of the predictive value of AMH. In addition, other factors such as stimulation protocols, laboratory environment variables (culture media, incubator systems), and patient-level characteristics (ethnicity, family disease history, major trauma history) were not included in our retrospective dataset.

Taken together, these limitations highlight the need for prospective, multicenter studies that incorporate both male-factor data and long-term outcomes such as live birth and miscarriage in order to more accurately determine the clinical utility of AMH beyond ovarian reserve assessment.

## 5. Conclusions

This study emphasizes the role of AMH as a reliable marker of ovarian reserve and oocyte yield in ICSI cycles, while showing limited predictive value for embryo quality and clinical pregnancy outcomes. Prognostic assessment in ART should therefore incorporate additional factors such as age, FSH levels, pronuclear count, embryo grade, and sperm quality. Prospective multicenter studies with long-term outcomes, including live birth and miscarriage, are warranted to further clarify the clinical utility of AMH beyond ovarian reserve evaluation.

## Figures and Tables

**Table 1:** Clinical, Oocyte, and Embryo Characteristics of Patients.

Parameter	Median	25%	75%
Age (years)	32	28	36
BMI	25.6	22.9	29.7
AMH	1.7	0.61	3.7
FSH	7.54	6.0	9.82
AFC	9	5	18
Total gonadotropin dose (IU)	2044	1550	2700
Number of retrieved oocytes	7	4	12
Number of mature oocytes	6	3	10
Number of ICSI oocytes	6	3	10
Oocyte quality index	5	4.6	5.5
Pronuclear count	3	1	5
Number of embryos transferred	1	0	1
Gr1 embryos	0	0	1
Gr2 embryos	0	0	1
Gr3 embryos	0	0	0
Gr4 embryos	0	0	0
Number of blastocysts	0	0	1

25–75%: 1st Quarter–3rd Quarter.

**Table 2:** Evaluation of Age, BMI, AMH, FSH, and AFC by Antral Follicle Count Category.

	Age	BMI	AMH	FSH	AFC
	Median (25–75%)				
Poor responder	35 (31–39)	25.9 (23–30.1)	0.45 (0.2–0.75)	10.1 (7.13–14.59)	3 (2–4)
Normo responder	32 (28–36)	25.2 (22.6–28.8)	1.33 (0.69–2.42)	7.45 (6.11–9.37)	9 (7–11)
Hyper responder	29 (25–32)	26.0 (23.1–30)	4.43 (2.99–7.00)	6.23 (5.24–7.42)	21 (17–30)
*p* *	<0.001 *	<0.001 **	<0.001 *	<0.001 *	<0.001 *

* Kruskal–Wallis H test. ** Anova test.

**Table 3:** Evaluation of Oocyte and Embryo Characteristics According to Antral Follicle Count Categories.

	Poor Responder	Normo Responder	Hyper Responder	*p*
	Median (25–75%)			
Total gonadotropin dose	2975 (2400–3450)	2175 (1800–2700)	1425 (1200–1750)	<0.001
Number of retrieved oocytes	3 (2–6)	7 (4–10)	13 (9–18)	<0.001 *
Number of mature oocytes	2 (1–5)	6 (3–8)	10 (6–14)	<0.001 *
Number of ICSI oocytes	3 (1–5)	6 (4–9)	10 (7–14)	<0.001 *
Oocyte quality index	5 (4.5–5.5)	5.1 (4.6–5.6)	5.0 (4.5–5.4)	0.012 **
Pronuclear count	1 (0–2)	3 (1–5)	5 (3–8)	<0.001 *
Number of embryos transferred	0 (0–1)	1 (0–1)	1 (0–1)	<0.001 *
Gr1 embryos	0 (0–1)	0 (0–1)	0 (0–1)	0.834
Gr2 embryos	0 (0–1)	0 (0–1)	0 (0–1)	0.627
Gr3 embryos	0 (0–0)	0 (0–0)	0 (0–0)	0.057
Gr4 embryos	0 (0–0)	0 (0–0)	0 (0–0)	0.280
Number of blastocysts	0 (0–1)	0 (0–1)	0 (0–1)	0.073

* Kruskal–Wallis H test. ** Anova test.

**Table 4:** Evaluation of Clinical, Oocyte, and Embryo Characteristics by IVF Outcome.

	No Pregnancy	Pregnancy	*p* *
	Median (25–75%)		
Age	32 (28–37)	30 (27–34)	<0.001
BMI	25.6 (22.9–29.7)	25.7 (22.9–29.2)	0.915
AMH	1.59 (0.57–3.54)	2.18 (0.87–4.13)	<0.001
FSH	7.64 (6.07–10.1)	7.20 (5.85–8.82)	<0.001
AFC	9 (4–18)	11 (6–22)	<0.001
Total gonadotropin dose administered for ovulation induction	2100 (1550–2750)	2025 (1550–2700)	0.127
Number of retrieved oocytes	7 (4–12)	9 (6–13)	<0.001
Number of mature oocytes	5 (2–9)	7 (4–10)	<0.001
Number of oocytes subjected to ICSI	6 (3–10)	7 (5–11)	<0.001
Oocyte quality index	5 (4.5–5.5)	5.2 (4.8–5.6)	<0.001
Pronuclear count	2 (1–5)	4 (3–6)	<0.001
Number of embryos transferred	1 (0–1)	1 (1–2)	<0.001
Gr1 embryos	0 (0–1)	1 (0–1)	<0.001
Gr2 embryos	0 (0–1)	0 (0–1)	0.784
Gr3 embryos	0 (0–0)	0 (0–0)	<0.001
Gr4 embryos	0 (0–0)	0 (0–0)	0.276
Number of blastocysts	0 (0–1)	0 (0–1)	0.429

* Mann–Whitney U test.

**Table 5:** Correlation Analysis of Clinical, Oocyte, and Embryo Characteristics by AMH Levels.

	Correlation Coefficient	*p* *
Age	−0.387	<0.001
BMI	−0.009	<0.001
FSH	0.806	<0.001
AFC	0.806	<0.001
Total gonadotropin dose administered for ovulation induction	−0.668	<0.001
Number of retrieved oocytes	0.695	<0.001
Number of mature oocytes	0.640	<0.001
Number of oocytes subjected to ICSI	0.656	<0.001
Oocyte quality index	−0.026	0.286
Pronuclear count	0.511	<0.001
Number of embryos transferred	0.145	<0.001
Gr1 embryos	0.021	0.457
Gr2 embryos	0.018	0.534
Gr3 embryos	−0.056	0.048
Gr4 embryos	−0.050	0.081
Number of blastocysts	0.142	<0.001

* Spearman correlation analysis.

**Table 6:** ROC Analysis of Clinical, Oocyte, and Embryo Characteristics for Predicting IVF Treatment Outcomes.

	AUC (CI)	*p*	Cut-Off Point	Sensitivite (%)	Spesifisite (%)
Age	0.565 (0.529–0.602)	0.001	31.5	52.2	40.8
BMI	0.495 (0.457–0.532)	0.781	25.6	49.3	52.7
AMH	0.489 (0.451–0.526)	0.553	1.78	49.2	50.8
FSH	0.547 (0.509–0.585)	0.015	7.54	52.3	47.7
AFC	0.472 (0.434–0.510)	0.146	9.5	49.6	53.6
Total gonadotropin dose administered for ovulation induction	0.527 (0.494–0.560)	0.128	2043	51.0	46.0
Number of retrieved oocytes	0.501 (0.464–0.538)	0.958	8.5	51.3	50.5
Number of mature oocytes	0.491 (0.454–0.529)	0.657	6.5	51.3	55.8
Number of oocytes subjected to ICSI	0.495 (0.458–0.532)	0.794	7.5	48.3	48.3
Oocyte quality index	0.475 (0.438–0.513)	0.202	5.16	46.9	52.3
Pronuclear count	0.451 (0.414–0.488)	0.012	3.5	50.0	57.3
Number of embryos transferred	0.481 (0.443–0.519)	0.322	1.5	25.3	29.6
Gr1 embryos	0.451 (0.413–0.489)	0.011	0.5	45.7	55.5
Gr2 embryos	0.486 (0.448–0.524)	0.464	0.5	42.6	45.5
Gr3 embryos	0.541 (0.504–0.578)	0.036	0.5	22.5	14.3
Gr4 embryos	0.502 (0.464–0.540)	0.902	0.5	3.1	2.2
Number of blastocysts	0.477 (0.439–0.516)	0.245	0.5	27.7	31.8

AUC: Area Under the Curve, CI: Confidence Interval.

**Table 7:** IVF Treatment Outcome Based on Patients’ Age, FSH, Pronuclear Count, Gr1 Embryo Count, and Gr3 Embryo Count.

	IVF Treatment Outcome	*p*
Age OR (95% GA)	0.062 (0.042–0.084)	0.001 *
FSH OR (95% GA)	0.072 (0.044–0.106)	0.001 *
Pronuclear Count OR (95% GA)	0.089 (0.063–0.118)	0.001 *
Gr1 embryos OR (95% GA)	0.372 (0.172–0.569)	0.001 *
Gr3 embryos OR (95% GA)	0.499 (0.175–0.887)	0.002 *

* Statistically significant (*p* < 0.05).

## Data Availability

The raw data supporting the conclusions of this article will be made available by the authors on request.
